# Effect of Bile Acid on Fetal Lung in Rat Model of Intrahepatic Cholestasis of Pregnancy

**DOI:** 10.1155/2014/308274

**Published:** 2014-03-23

**Authors:** Ling Yu, Yiling Ding, Ting Huang, Xiaoxia Huang

**Affiliations:** Department of Obstetrics and Gynecology, The Second Xiangya Hospital of Central South University, 139 Renmin Zhong Lu, Changsha, Hunan 410011, China

## Abstract

*Objective.* To determine the correlation between maternal bile acid (BA) level and fetal pulmonary surfactant in rats and study the effects of BA on fetal lung in rat model of intrahepatic cholestasis of pregnancy. * Methods*. Forty pregnant rats were treated with (A) 5.5 mg/kg BA, (B) 1.4 mg/kg BA, and (C) 1 ml physiological saline. Levels of total bile acid (TBA), ALT, AST, TBIL, DBIL, and SP-A were determined and the lungs of fetal rats were analyzed for pathological changes. * Results*. Groups A and B intervened with BA showed significant higher level of TBA in both maternal and fetal serum, more mortality rate of fetal rats, more concentration of SP-A in fetal serum, and wider alveolus mesenchyme of fetal rats than the control Group C. Higher level of BA associated with increased fetal risk and lower numerical density of mitochondria in type II alveolar epithelial cells. The levels of TBA in maternal serum were found to have significant positive correlation with those in fetal serum and SP-A level but negatively with the area of alveolus and the numerical density of lamellar body. * Conclusions*. The TBA level in maternal serum showed significant association with lung pathological changes in fetal rats.

## 1. Introduction

Intrahepatic cholestasis of pregnancy (ICP) is liver disease which could lead to premature birth, fetal distress and neonatal asphyxia, and increasing risk of fetal morbidity and mortality [1]. The incidence of ICP varies throughout the world from 0.4% to 15% [2, 3]. The best biomarker for diagnosis and follow-up of ICP is up to knowing percentage levels of bile acids (taurocholic and glycocholic acids) over 40% with TBA ≥14 mmol/L. The level of bile acid is found to be associated with fetal complications [1, 4]. Howard and Murphy found that fetal serum TBA was higher than that of the maternal level during late stage of normal pregnancy [4]. Bile acid is transferred from fetus to mother through fetus circulation, and then mother exhausted the exceeding bile acid out of the body with normal liver and gall system. While ICP occurs, high bile acid level in maternal blood made damage to placental transport, leading to bile acid deposition in fetal body [5].

Recently, the abnormalities of pulmonary surfactant system have been implicated in the pathogenesis of respiratory distress syndrome [6, 7]. However, whether the level of TBA in maternal serum caused perinatal abnormality of pulmonary surfactant and fetal lung tissue morphological structure remains largely unknown. Recently, bile acid was found to cause surfactant inactivation by enhancing the activity of secretory phospholipase A2 (sPLA2) and induce inflammatory response of fetal lung [5, 8]. Surfactant protein A (SP-A) is a main inhibitor of sPLA2 and serves as an integral component of alveolar surfactant consisting of 85~90% phosphatides, 5~10% protein, and 5% sugar [9]. SP-A is covered on the alveolus surface to decrease the surface tension, thus prevent alveolus collapse, and stabilize pulmonary alveolar pressure [9]. Phospholipids include phosphatidylcholine (PC), sphingomyelin (SM), inositol lipid (PI), and lysophosphatidyl choline (LPC), among which LPC is the main component of oxidized low density lipoprotein (LDL) and degrades production of PC with its cytotoxic effect [10]. SP-A acts as a pulmonary surfactant binding protein and is secreted by type II pneumonocyte. When the lung is damaged, the level of SP-A is increased through permeability augmented alveolus-capillary membrane and released to blood circulation. Some groups reported SP-A in serum as a tool to evaluate the integrality of alveolus-capillary membrane and the degree of lung injury [11, 12].

However, some bile acids have toxic effects on a variety of cells. For example, chenodeoxycholic and lithocholic acids are cytotoxic and have detergent effects.* In vitro* experiments showed that chenodeoxycholic acids could also disrupt cellular membranes in the alveoli, increasing cationic permeability and intracellular Ca^2+^ concentration, leading to overloading of Ca^2+^ in cells, and causing injury to type II pneumonocyte [13]. Porembka et al. reported that bile acid aspiration induced severe chemical pneumonitis in a porcine lung model [14]. Intratracheally injection of bile acid had been shown to induce severe pulmonary edema in rabbits [15]. Toxic effect of high concentrations of bile acid may influence the structure and function of multiple fetal organs, closely correlated with the poor prognosis of fetuses [16]. Recently, we integrated the method of Tian and Sun [17] and Qixin et al. [18] by directly injecting 5.5 mg/kg and 1.4 mg/kg to pregnant rats intraperitoneally with bile acid for 8 days and successfully constructed the maternal high dose bile acid model [16, 19]. In order to determine the correlation between maternal TBA level and fetal pulmonary surfactant and study the effects of bile acid on fetal lung in rat model of ICP, in this present study, we measured the TBA level and SP-A in the serum of fetal rats from their mothers with ICP and analyzed the effect of high maternal bile acid level on perinatal pulmonary function disorder in fetal rats.

## 2. Materials and Methods

### 2.1. Animals

Forty Sprague-Dawley pregnant rats were randomly divided into three groups: Group A, Group B, and Group C. The rats provided by animal experiment center of Xiangya Hospital, Central South University, were weighed 250~270 g and aged at 150~180 days. From day 13 of gestation, rats in Group A and Group B were injected intraperitoneally with bile acid (cholic acid (CA), Sigma-Aldrich, USA) for 8 days consecutively. Group A (high dose group) was injected with 5.5 mg/kg bile acid. Group B (low dose group) was injected with 1.4 mg/kg bile acid. Group C (experimental control group) was injected intraperitoneally with Sodium Chloride (NS). On day 13 of gestation, 1 mL blood was drawn from tail vein of pregnant rats to determine the levels of TBA, alanine aminotransferase (ALT), aspartate aminotransferase (AST), total bilirubin (TBIL), and direct bilirubin (DBIL). On day 21 of gestation, cesarean section was performed with anesthesia by intraperitoneal injection of 10% hydral with dosage of 3 mL/kg and the number of fetal rats, the number of still birth, and fetal weight were recorded. 1 mL blood from abdominal vein of pregnant rats was drawn to measure the levels of TBA, ALT, AST, TBIL, and DBIL. The fetal rat serum was collected to determine the level of TBA and SP-A. Ten fetal rats were selected randomly and the lung tissue was completely removed with ophthalmic scissors. The tissue was cut in size of about 1 mm × 1 mm × 1 mm from the lower right lung and then put in 2.5% glutaraldehyde phosphate buffer to fix specimens used for the electron microscopy. The remaining lung tissue was fixed by 10% formalin to be used for light microscope analysis.

### 2.2. Biochemical Analysis

**Figure 1:**
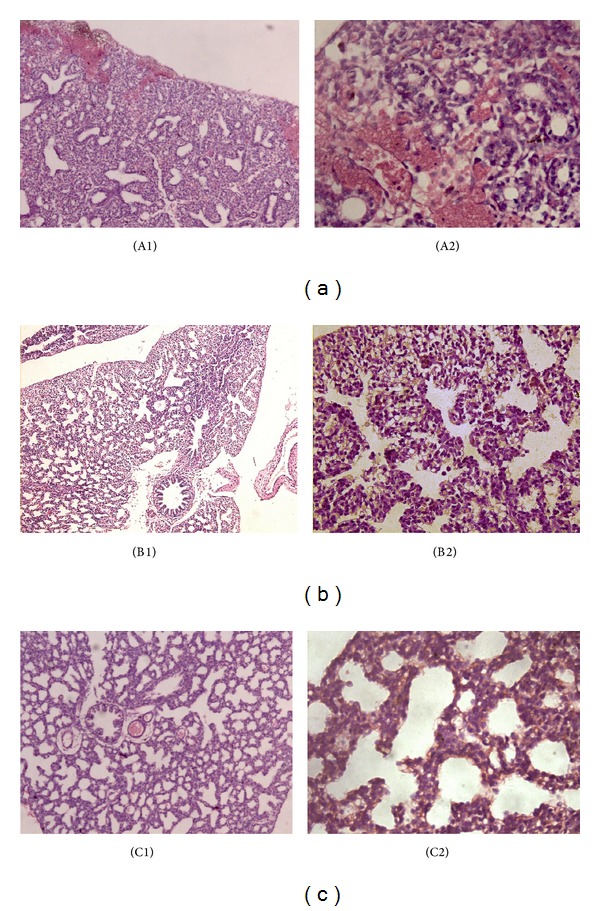
The morphology of fetal rat lungs in light microscope. (a) The high bile acid group: immature fetal lung tissue with smaller alveolar space, thicker alveolar septal, and a large number of hemorrhagic lesions were observed. HE: ×100 (A1), ×400 (A2). (b) The low bile acid group: fetal lung tissue with small alveolar space and thick alveolar septal were observed. HE: ×100 (B1), ×400 (B2). (c) The experimental control group: mature fetal lung tissue with large alveolar space and thin alveolar septal were observed. Alveolar epithelial cells, cytoplasm stained (red), and nuclear (flat). HE: ×100 (C1), ×400 (C2).

**Figure 2:**
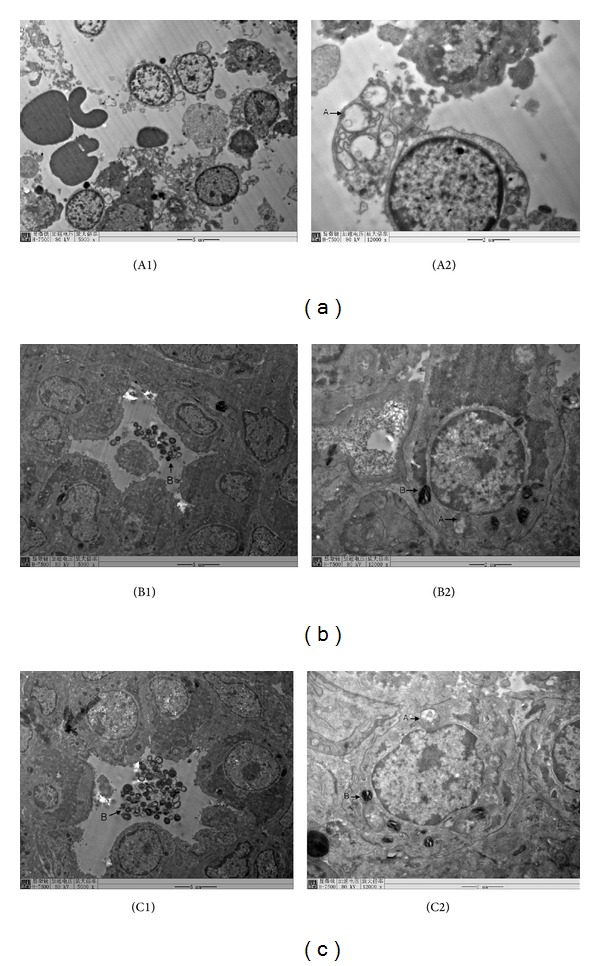
The morphology of fetal rat lungs in electronic microscope. (a) The high bile acid group: type II alveolar epithelial cells with swelling mitochondria were degenerated. Lamellar body had disappeared. A large number of red blood cells, proteins, cell debris falling, and edema fluid were filled in alveolar cavity. ×5000 (A1), ×12000 (A2). (b) The low bile acid group: type II alveolar epithelial cells with decreasing microvilli on the surfaces and slight swelling mitochondria were observed. ×5000 (B1), ×12000 (B2). (c) The experimental control group: type II alveolar epithelial cells were in good condition. A clear lamellar structure can be observed in lamellar bodies. ×5000 (C1), ×12000 (C2). Arrow A: swelled mitochondria (balloon-like); arrow B: lamellar body.

Hitachi 7060 Automatic Biochemistry Analyzer was used to measure TBA, ALT, AST, TBIL, and DBIL and amniotic fluid TBA. The concentrations of SP-A in the fetal rat blood were investigated by ELISA (US Science and Technology Co., Ltd., China). High efficiency liquid chromatography (HPLC) was applied to determine the contents of amniotic fluid phosphatidyl choline (PC), phosphatidyl inositol (PI), lysophosphatidyl choline (LPC), and sphingomyelin (SM), which were provided by Sigma-Aldrich (Shanghai, China). Pure grade methanol, phosphoric acid, chloroform and acetonitrile were purchased from Medicines Group Shanghai Chemical Reagent Co., Ltd. HPLC system was manufactured by Shimadzu Corporation. Hw-2000 color spectrum data station was provided by Nanjing Qianpu Software Corporation. The main procedures were described as follows: 5 mg of standard phospholipids product was accurately weighed and solved by 100 mL chloroform-methanol and mixed evenly; phospholipids in the taken samples were solved by 10 mL chloroform-methanol. Under the above color spectrum conditions, 20**μ**L standard solution and sample solution were taken and chromatograms of the standard sample and experimental sample were obtained (Figures 1 and 2). Lung tissue samples were removed from 10% formaldehyde solution to the gradient alcohol dehydration, paraffin-embedded, sections (thickness 4 *μ*m), and hematoxylin-eosin (HE) staining. Lung tissue samples were removed from fixed specimens fixed by 2.5% glutaraldehyde phosphate buffer and then by 1% osmic acid fixation, gradient acetone dehydration, Epon812 embedded agent, and ultrathin sections (thickness 60 nm), as uranium and lead citrate double staining, then observed under Hitachi H-7500 transmission electron microscope and photographed with Germany CCD camera system.

### 2.3. Morphology of Fetal Lung

HE stained sections were put under the optical microscope to observe morphological development of fetal lung. 6 fields of vision were randomly selected from each slice and analyzed by the image analysis system to determine alveolar septum thickness and alveolar size [20]. The alveolar type II cells were measured by transmission electron microscope at high power field (X1200), and cube, large nuclear, round dense chromatin, the microsurface short villi, and the lamellar bodies were identified. Five photos were taken for each ultrathin section; the mitochondria and lamellar body were observed. Photoshop 8.0 software was used to develop the test network, the size of which is 120 mm × 90 mm. The point distance is 1 mm, and there are a total of 10,800 test points in the network. Calculation formula was referred to the method described in previous literature [21]: $Nv=\left(M^{3}/10^{9}\right)\times {\left[\sum Nx/(d^{2}\times \sum Pc)\right]}^{3/2};\;\;\overset{-}{V}=(\sum Px/\sum Pc)/Nv$.

### 2.4. Statistics

SPSS13.0 software was adopted to proceed statistical analysis, and the results were presented as $\text{mean}\pm \text{S}.\text{D}.\;(\overset{-}{x}\pm s)$; one-way ANOVA was used to proceed multiple groups of data; LSD *t*-test was used to compare between groups of multiple groups, *χ* ^2^ test was used to compare the rates of different samples, and Spearman correlation analysis was applied to do correlation analysis.

## 3. Results

### 3.1. Comparison of the Biochemical Indicator Levels in Maternal Serum of Three Groups before Intervention

There were no significant differences in TBA concentration of maternal serum between Group A (3.45 ± 1.74 *μ*mol/L), Group B (3.32 ± 1.90 *μ*mol/L), and Group C (3.53 ± 1.97 *μ*mol/L), *P* > 0.05. No significant differences were found in TBA, ALT, AST, TBIL, and DBIL between Group A, Group B, and Group C before intervention, *P* > 0.05, as shown in Table 1.

### 3.2. Comparison of Biochemical Indicator Levels in Maternal Serums of Three Groups after Intervention

The level of TBA in maternal serum of Group A (21.09 ± 8.58 *μ*mol/L) was significantly higher than that of Group B (10.49 ± 3.22 *μ*mol/L), *P* < 0.01; the level of TBA in Group B (3.98 ± 2.18 *μ*mol/L) was significantly higher than that in Group C, *P* < 0.01, as shown in Table 2.

### 3.3. Comparison of TBA and SP-A in Fetal Serum of the Three Groups after Intervention

The level of TBA in fetal serum of Group A (26.3 ± 11.00) *μ*mol/L was significantly higher than that in Group B (10.73 ± 4.76) *μ*mol/L, *P* < 0.01; the TBA level in Group B was significantly higher than that in Group C (4.08 ± 2.40) *μ*mol/L, *P* < 0.05. The concentration of SP-A in fetal serum of Group A (12.45 ± 4.67 ng/mL) was significantly higher than that in Group B (7.33 ± 2.54 ng/mL), *P* < 0.01; the concentration of SP-A in Group B was also significantly higher than that in Group C (2.71 ± 1.30 ng/mL), *P* < 0.01, as shown in Table 3. Correlation analysis showed that TBA levels in maternal serums were significantly positively correlated with those in fetal serum (*r* = 0.882, *P* < 0.01), and the level of SP-A is also significantly positively correlated with the level of TBA (*r* = 0.708, *P* < 0.01) (Table 3).

### 3.4. Comparison of the Growth and Development of Fetal Rats in Three Groups

The mortality rate of fetal rats in Group A (18.35%) was higher than that in Group B (8.82%), *P* < 0.01; the mortality rate of fetal rats in Group B was significantly higher than that in Group C (3.77%), *P* < 0.01, as shown in Table 4.

### 3.5. Pathological Changes of Fetal Rat Lungs of Three Groups under Light Microscopy

In the experimental control group, mature fetal lung tissues were characterized with large alveolar space and thin alveolar septal, and the alveolar epithelial cells were found to be arranged (cytoplasm in red, nuclear in flat) (Figure 1(b)). In low dose group, normal structure of fetal type II pneumonocyte was observed and microvillus outstanding cell surface fell off a little with clear cellular nuclear structure. Mitochondria had slight swelling, enlarging volume, degenerated vacuolar, and disrupted or decreased crista. The number of lamellar body in alveolar space and alveolus type II cell endochylema reduced, with some demonstrating vacuolization (Figure 1(c)). For high dose group, immature fetal lung tissues with smaller alveolar space and thicker alveolar septal were observed. A large number of hemorrhagic lesions were observed. It also showed that the area of alveolus of fetal rats in Group A (286.15 ± 26.38 *μ*m^2^) was significantly smaller than that in Group B (717.46 ± 22.07 *μ*m^2^) (*P* < 0.01); the area of alveolus of fetal rats in Group B was significantly smaller than that in Group C (900.13 ± 15.89 *μ*m^2^), *P* < 0.01. The width of alveolus mesenchyme of fetal rats in Group A (70.21 ± 8.95 *μ*m) was significantly thicker than that in Group B (32.21 ± 4.23 *μ*m) (*P* < 0.01), while the width in Group B was thicker than that in Group C (23.51 ± 2.73 *μ*m), *P* < 0.01, as shown in Table 5. Correlation analysis showed that the area of alveolus was significantly negatively correlated with the levels of TBA (*r* = −0.794, *P* < 0.01) and SP-A (*r* = −0.734, *P* < 0.01) (Table 5).

### 3.6. Ultrastructural Observation and Stereoscopy Analysis of Fetal Rat Lung Tissue in Three Groups

In the experimental control group, type II alveolar epithelium had clear structure, with short microvillus on its surface; the nucleus was oval and the karyolemma was complete, with uniform distribution of chromatin. Abundant lamellar body could be observed in alveolar and type II intracytoplasm, which was in dark color and having clear lamellar structure (Figure 2(c)). In low dose group, normal structure of fetal type II pneumonocyte was observed and microvillus outstanding cell surface fell off a little with clear cellular nuclear structure. Mitochondria had slight swelling, enlarging volume, degenerated vacuolar, and disrupted or decreased crista. The number of lamellar body in alveolar space and alveolus type II cell endochylema reduced, with some demonstrating vacuolization (Figure 2(b)). In the high dose group, type II alveolar epithelial cells denatured and died, with disappearance of microvillus structure on cell surface and swelling mitochondria. Mitochondria swelled seriously, showing balloon-like change and crista cavitation vanished. Lamellar body numbers reduced significantly and the lamellar structure disappeared. A large number of red blood cells, proteins, cell debris falling, and edema fluid are filled in alveolar cavity (Figure 2(a)).

Comparison of mitochondria density and average volume of type II alveolar cells in fetal rats of four groups showed that the mitochondria density intype II alveolar cells ofGroup A (20.44 ± 4.99 *μ*m^−3^) was significantly lower than that of Group B (31.46 ± 3.45 *μ*m^−3^), *P* < 0.01; the density between Group B and Group C had no statistical difference, *P* > 0.05. Comparison of the average volume of mitochondria between the two dose groups (23.22 ± 2.76 × 10^−3^ *μ*m^3^and 21.27 ± 2.62 × 10^−3^ *μm* ^3^) showed no significant difference. However, these two dose groups had significantly higher average volume of mitochondria than the control, *P* < 0.01.

Comparison of the density and average volume of lamellar body in fetal rats between four groups showed that Group A (47.98 ± 3.88 *μ*m^−3^  and 12.22 ± 2.89 × 10^−3^ *μ*m^3^) was lower than Group B (74.46 ± 3.06 *μ*m^−3^  and 23.27 ± 2.43 × 10^−3^ *μ*m^3^). Group B was significantly lower than Group C (81.30 ± 6.56 *μ*m^−3^ and 31.40 ± 2.92 × 10^−3^ *μ*m^−3^), as shown in Table 6. There is significant negative correlation between the numerical density of lamellar body and SP-A (*r* = −0.701, *P* < 0.01).

## 4. Discussion

Cholic acid, one of the bile acid species we used in this study, may have toxic effects on a variety of cells. In this study, our animal studies showed that there is no significant difference in the concentration of TBA, ALT, AST, TBIL, and DBIL between the three groups before intervention. However, after intervention, the level of TBA in maternal serum of Group A is significantly higher than that in Group B, and Group B shows significantly higher than that in the control Group C. Further, there is no significant difference in the concentration of ALT, AST, TBIL, and DBIL among these four groups. It showed that both of the two doses of bile acid (5.5 mg/kg, 1.4 mg/kg) could cause increasing maternal serum bile acid level without increasing bilirubin level, thus excluding the toxic effects of bilirubin in this study.

Bile acid could enter lung tissue by two ways in terms of amniotic fluid aspiration and blood circulation. Previous studies have showed that high concentration of bile acid gathered in alveolus cleaning solution in ICP neonates [22], but the incidence of ICP in neonatus had no direct correlation with bile acid concentration in amniotic fluid, while it had close relationship with high bile acid level in fetal blood [2, 23]. Our results demonstrate that (1) bile acid concentration of fetal rats serum in dose group was significantly higher than that of the experimental group and blank control group, and there is positive correlation between TBA of pregnant rat serum and that of fetal rat serum; (2) the still birth rate in dose groups was significantly higher than that of the experimental group and blank control group. These results suggest the close correlation between bile acid concentration and prognosis of the fetus. “Bile Acid Pneumonia” had been first reported by Zecca et al. [3] and paved a way for analysis of ICP. Some clinical studies further indicated the role of bile acids in determining respiratory failure in neonates. Excluding the fetal age, the incidence of RDS in newborns from cholestatic pregnancies is twice that of control population (28.6% versus 14%) and it shows positive correlation with TBA level in maternal serum [24, 25]. It has been reported that the most important pathogenesis of respiratory distress syndrome was the abnormality of pulmonary surfactant [6, 10].

Pulmonary surfactant is secreted by type II alveolar epithelial cells, which can reduce tension in the surface of alveolar in order to maintain the alveolar stability. De Luca et al. showed that bile acids cause surfactant inactivation by enhancing the activity of secretory phospholipase A2 (sPLA2), a key enzyme in the lung injury pathway [8]. Herraez et al. further confirmed the sPLA2 mechanisms and showed the accumulation of bile acids into the fetal rat lung [5]. Previously, SPA-A was shown as a main inhibitor of sPLA2 and a major component of pulmonary surfactant [9]. Under normal circumstance a small number of SP-A leak into the blood through alveolar epithelium. SP-A is specific to the lung, and a lot of SP-A leak into the circulation of patients with acute respiratory distress syndrome in a manner inversely related to lung function. So serum SP-A is an acute indicator of lung function and alveolus-capillary membrane injury [11, 13]. In addition, emerging evidences have shown that anti-inflammatory proteins like SP-A or Clara protein (CCSP) that inhibit sPLA2 increase in response to lung injury in order to lower inflammation and reduce lung injury. For example, De Luca et al. showed that CCSP significantly increased during meconium aspiration syndrome which is rich of bile acids [25]. Consistently, our results showed that the concentration of SP-A in fetal serum in bile acid groups is higher than that in control groups. The level of SP-A shows significantly positive correlation with the level of TBA but negative correlation with the area of alveolus. The result that level of SP-A increases in fetal rat serums is consistent with damaging effects of bile acid on the lung epithelium, and the destruction of alveolocapillary membrane [26].

Interestingly, immature fetal lung tissues with smaller alveolar space and thicker alveolar septal were found in high bile acid group. A large number of hemorrhagic lesions were also observed. The width of alveolus mesenchyme of fetal rats in bile acid group was thicker than that in control groups. There is negative correlation between the area of alveolus and TBA. The thinning of the alveolar space and the expansion of alveolar area are the morphologic sign of mature lungs [27]. We observed that the morphology of fetal lung was damaged in bile acid group, characterized as a small alveolar and a thick alveolar septal. This morphology would affect gas exchange function in the fetus after its birth.

In addition, type II alveolar epithelial cells with swelling mitochondria were degenerated in high bile acid group, and lamellar body disappeared. A large number of red blood cells, proteins, cell debris falling, and edema fluid were filled in alveolar cavity. In low bile acid group, type II alveolar epithelial cells with decreasing microvilli on the surfaces and slight swelling mitochondria were observed. The density and average volume of lamellar body ofbile acid group were lower than those in control group. There is significant negative correlation between the density of mitochondria intype II alveolar cells and TBA. There is significant negative correlation between the density of lamellar body and SP-A. It may result from the damaging effects of bile acid on the lung epithelium. Lamellar body is a distinctive structure in type II alveolar epithelial cells. Because of the degeneration and necrosis of type II alveolar epithelial cells, the synthesis of pulmonary surfactant was hampered and affected by the perinatal prognosis.

In summary, we found that, as an increasing bile acid in the maternal blood, the morphology of fetal lung tissue and alveolar-capillary membrane are disposed to be damaged. Some changes in the mitochondria of type II alveolar cells and lamellar body were observed in our study. The necrosis of type II alveolar cells and a decreasing of lamellar body may lead to hampering of the synthesis of pulmonary surfactant, which may impair normal breathe of fetus at birth and affect the perinatal prognosis.

## Figures and Tables

**Table 1:** Comparison of biochemical indicator level in maternal serum of three groups before intervention ($\overset{-}{x}\pm s$).

Group	*N*	TBA	ALT	AST	TBIL	DBIL
		(*µ*mol/L)	(U/L)	(U/L)	(*µ*mol/L)	(*µ*mol/L)
A	10	3.45 ± 1.74	38.97 ± 8.31	129.01 ± 19.36	0.08 ± 0.10	0.02 ± 0.04
B	10	3.32 ± 1.90	40.44 ± 5.89	134.43 ± 24.19	0.07 ± 0.11	0.03 ± 0.07
C	10	3.53 ± 1.97	41.36 ± 7.57	141.37 ± 38.26	0.03 ± 0.07	0.01 ± 0.03
*P *		0.924	0.818	0.521	0.545	0.830

**Table 2:** Comparison of biochemical indicator levels in maternal serum of three groups after intervention ($\overset{-}{x}\pm s$).

Group	*N*	TBA	ALT	AST	TBIL	DBIL
		(*µ*mol/L)	(U/L)	(U/L)	(*µ*mol/L)	(*µ*mol/L)
A	10	21.09 ± 8.58^△^	42.39 ± 6.24	149.29 ± 37.02	0.07 ± 0.08	0.04 ± 0.07
B	10	10.49 ± 3.22*	44.34 ± 7.24	148.07 ± 31.96	0.08 ± 0.11	0.03 ± 0.05
C	10	3.98 ± 2.18^#^	42.87 ± 7.21	148.52 ± 36.04	0.05 ± 0.08	0.02 ± 0.04
*P *		<0.01	0.866	0.956	0.574	0.578

Note: ^△,∗^compared with the control, *P* < 0.01; ^△,∗,#^compared with each other, *P* < 0.01.

**Table 3:** The levels of TBA and SP-A in fetal serum of three groups $(\overset{-}{x}\pm s$).

Group	*N*	TBA in maternal serum (µmol/L)	TBA in fetal serum (µmol/L)	SP-A in fetal serum (ng/mL)
A	10	21.09 ± 8.58	26.3 ± 11.00^△^	12.45 ± 4.67^△^
B	10	10.49 ± 3.22	10.73 ± 4.76*	7.33 ± 2.54*
C	10	3.98 ± 2.18	4.08 ± 2.40^#^	2.71 ± 1.30^#^
*P *		<0.01	<0.01	<0.01

Note: ^△,∗^compared with the control, *P* < 0.01; ^△,∗,#^compared with each other, *P* < 0.01.

**Table 4:** Comparison of the growth and development of fetal rats in three groups.

Group	*N*	Total fetus	Death fetus	The mortality rate of fetal rats	Average weight
A	10	109	20	18.35^△^	4.01 ± 0.21^△^
B	10	102	9	8.82*	4.62 ± 0.41*
C	10	106	4	3.77^#^	5.14 ± 0.48^#^

Note: ^△,∗^compared with the control, *P* < 0.01; ^△,∗,#^compared with each other, *P* < 0.01.

**Table 5:** Comparison of morphological development of fetal rat lungs in three groups $(\overset{-}{x}\pm s$).

Group	*N*	The width of alveolus mesenchyme (*µ*m)	The area of alveolus (*µ*m^2^)
A	10	70.21 ± 8.95^△^	286.15 ± 26.38^△^
B	10	32.21 ± 4.23*	717.46 ± 22.07*
C	10	23.51 ± 2.73^#^	900.13 ± 15.89^#^
*P *		<0.01	<0.01

Note: ^△,∗^compared with the control, *P* < 0.01; ^△,∗,#^compared with each other, *P* < 0.01.

**Table 6:** Comparison of mitochondria and stereology of lamellar body in type II epithelium in fetal rats of three groups ($\overset{-}{x}\pm s)$.

Group	*N*	Mitochondria		Lamellar body	
		Number density	Average volume	Number density	Average volume
		(*µ*m^−3^)	(×10^−3^ *µ*m^3^)	(*µ*m^−3^)	(×10^−3^ *µ*m^3^)
A	10	20.44 ± 4.99^△△△^	23.22 ± 2.76^△△^	47.98 ± 3.88^△^	12.22 ± 2.89^△^
B	10	31.46 ± 3.45	21.27 ± 2.62^△△^	74.46 ± 3.06*	23.27 ± 2.43*
C	10	32.84 ± 3.17	15.02 ± 2.91	81.30 ± 6.56^#^	31.40 ± 2.92^#^
*P *		<0.01	<0.01	<0.01	<0.01

Note: ^△△△,△△,△,∗^compared with the control, *P* < 0.01; ^△,∗,#^compared with each other, *P* < 0.01.
